# Robot location privacy protection based on Q-learning particle swarm optimization algorithm in mobile crowdsensing

**DOI:** 10.3389/fnbot.2022.981390

**Published:** 2022-09-30

**Authors:** Dandan Ma, Dequan Kong, Xiaowei Chen, Lingyu Zhang, Mingrun Yuan

**Affiliations:** School of Information and Electronic Technology, Jiamusi University, Jiamusi, China

**Keywords:** crowdsensing service, Q-learning, particle swarm optimization, location privacy protection, RLBS

## Abstract

In the recent years, with the rapid development of science and technology, robot location-based service (RLBS) has become the main application service on mobile intelligent devices. When people use location services, it generates a large amount of location data with real location information. If a malicious third party gets this location information, it will cause the risk of location-related privacy disclosure for users. The wide application of crowdsensing service has brought about the leakage of personal privacy. However, the existing privacy protection strategies cannot adapt to the crowdsensing environment. In this paper, we propose a novel location privacy protection based on the Q-learning particle swarm optimization algorithm in mobile crowdsensing. By generalizing tasks, this new algorithm makes the attacker unable to distinguish the specific tasks completed by users, cuts off the association between users and tasks, and protects users' location privacy. The strategy uses Q-learning to continuously combine different confounding tasks and train a confounding task scheme that can output the lowest rejection rate. The Q-learning method is improved by particle swarm optimization algorithm, which improves the optimization ability of the method. Experimental results show that this scheme has good performance in privacy budget error, availability, and cloud timeliness and greatly improves the security of user location data. In terms of inhibition ratio, the value is close to the optimal value.

## Introduction

Robot location-based service (RLBS) system based on crowdsensing through the interactive of physical space and information space can be flexible, efficiently acquire, and transmit all kinds of scene data. In addition, through the communication network, the collected perception data are transmitted to the server for intelligent processing, so as to provide customized personalized, real-time location awareness service for users. On the one hand, due to the security vulnerabilities of wireless sensor network, mobile communication network, mobile terminal equipment, RFID tag and other technologies, and equipment, perception data in the process of collection and network transmission are facing security threats such as illegal monitoring, interference, theft, and modification. On the other hand, most RLBS systems can only mine and analyze the plain-text data after decryption during the storage and intelligent processing of perceptual data on the server side, which gives attackers and illegal RLBS service providers the opportunity to obtain, sell, and use the private data on the server side. In particular, the current application environment of robots is very wide, so it is very important to protect the location privacy of robots. Because the situation awareness data of RLBS service in the Internet of Things (IoT) space not only contain privacy information such as robot identity, bank account, current location, and activity track, but also may involve business secrets such as enterprise marketing plan, product information, and customer information. Compared with the previous Internet-based RLBS system, the RLBS system in the IoT space faces more severe privacy security problems. If the privacy security of LBS service in the IoT space cannot be guaranteed, the popularity of RLBS service in the field of robot application and large-scale promotion in the commercial field will be seriously affected and even bring catastrophic losses and consequences.

Along with the development of the Internet, smart phones, tablets, wearable devices, automotive sensors, and other mobile terminals integrate more and more sensors. The public has more and more powerful computing, sensing, storage, and communication abilities (Gupta et al., 2020; Laghari et al., 2020; Rui et al., 2021). Using these abilities reasonably through some incentive measures, many environmental perception problems can be solved cheaply and efficiently (Liu R. et al., 2019).

Crowdsensing applications inspire users to perceive data by releasing perception tasks, then analyze, and use these data. However, this new approach also raises new privacy concerns. Once a user completes a sensing task, the crowdsensing service provider can infer that the user appears in the data collection range of the task during the task collection period (Dong et al., 2022), so as to master the user's behavior pattern using the trajectory of the user completing the task. When the user's privacy is threatened, the user will lose the initiative to participate in the group intelligence awareness application.

As long as the users utilize the location service, the attacker can deduce when and where the user went according to the real-time location information of the user. The sensitive location such as the home address and work place will also be exposed. The health status of users and living habits can be inferred by attackers. Therefore, how to protect users' location privacy has always been an important issue concerned by the researchers (Wang et al., 2021).

With the increasing demand for computer storage and computing, the traditional location privacy protection model based on cloud computing becomes increasingly overwhelmed in performance and security. Compared with cloud computing (Ademaj and Bernhard, 2021), fog computing (Karthik and Kavithamani, 2021) adopts a more distributed architecture that is closer to the edge of the network. Fog computing centralizes data, data processing, and applications in devices at the edge of the network, rather than keeping them almost all in the cloud. Fog computing can not only solve the problem of networked devices automation. More importantly, it requires less data transmission, which is conducive to improving local storage and computing capacity and eliminating the bottleneck of data storage and data transmission (Song et al., 2022).

Traditional location privacy protection technologies include space concealment (Anh et al., 2010; Liu et al., 2021), location offset and blurring (Freudiger et al., 2013; Pournaras et al., 2016), and forging false location (Gao et al., 2022), etc. However, these technologies require forgery or modification of data acquisition location or time, which will affect the availability of crowdsensing task data. Existing privacy protection technologies in crowdsensing environment mainly rely on k-anonymization (Laohakiat and Sa-Ing, 2021) and other methods to generalize users. However, the crowdsensing incentive mechanism needs to reward the task completers, and the generalization of users will affect the operation of the incentive mechanism.

In this paper, a privacy protection strategy for generalized task is designed to avoid the influence of privacy protection mechanism on incentive mechanism. To ensure the availability of data, the confusion task adopts real tasks performed by other users. When there are not enough users to participate in the generalization and the system cannot meet the privacy protection requirements of anonymity, the suppression method is adopted to give up the task. When users submit sensing tasks continuously, different generalization task sets will affect the possibility of the next task being suppressed. Our main contributions are as follows. To reduce the inhibition rate, we propose a Q-learning particle swarm optimization algorithm in mobile crowdsensing, which continuously tries different combinations of confounding tasks and trains a confounding task selection scheme that can output the lowest inhibition rate. In addition, we use this network to make decisions about confusing task selection. Experimental results show that the privacy protection strategy in this paper can protect users' location privacy with low inhibition rate without destroying the effectiveness of sensing task.

This paper is organized as follows. In Section 2, we give the Related works for this paper. Section 3 introduces the Task characteristics and system model. In Section 4, the Task decision problem for minimizing inhibition rate is described. Task decision-making scheme based on Q-learning particle swarm optimization is explained in Section 5. Experiments and analysis are displayed in Section 6. There is a Conclusion in Section 7.

## Related works

Usually, the tasks posted by crowdsensing servers are time-sensitive and location-sensitive. There is a crowdsensing task, and it requires to sense the noise data of a residential area at 10 PM. The noise data uploaded by the user at 9 o 'clock in the area or nearby area are invalid. Traditional privacy protection technologies often distort time or generalize location to protect users' privacy. These methods will destroy the validity of the data in the crowdsensing environment. Therefore, facing the time-sensitivity and location-sensitivity of crowdsensing tasks, how to protect users' privacy without destroying the availability of task data is an urgent problem to be solved.

Common privacy protection technologies in crowdsensing environment mainly include confusion zone technology, third-party anonymity technology, k-anonymity technology, and suppression technology, etc.

Wang et al. (2013) allowed users to set personalized privacy requirements and then sent sensing data by randomly expanding a confusion zone according to the users' privacy requirements through the confusion algorithm. This method was vulnerable to location inference attacks when users submitted sensing data continuously. Moreover, due to the location sensitivity of sensing tasks, sending sensing data in a confused region would cause data invalidation.

Hare et al. (2018) performed random number anonymous binding for task and its own feature data through a third-party anonymous server. The server calculated the similarity of the uploaded perception data and feeded back the reputation value, so that the server could not obtain the user's characteristic information and sensing information at the same time. It also limited the user's behavior through reputation. However, it increased the communication overhead and the risk of privacy leakage from third-party servers.

Wu et al. (2019) introduced a privacy protection technology based on k-anonymity, which generalized users and made the server unable to distinguish which user in the k users had completed the crowdsensing task, thus protecting users' privacy. The core idea was to generate a two-dimensional space (tile) identity card (ID) instead of their real location when users uploaded sensing data. The tile space was expanded horizontally and vertically until the number of users was ≥1. However, if users continuously submitted sensing tasks, an anonymous set of K different users A1, A2, … An was generated. The attacker could quickly locate the real users by $\underset{i=1}{\overset{n}{\cap }}A_{i}$ and the scheme generalizes the users who complete the task, affecting the operation of the knowledge incentive mechanism of crowdsensing.

Yang and Jiang (2021) developed a novel region query framework that could provide robust privacy for location-dependent queries. Then, an oblivious transfer-assist privacy-aware protocol was designed for location-based service with rigorous security analysis. However, this protection method focused excessively on the location attribute and ignored the content attribute contained in the RLBS, which disclosed the user's private information. Liu et al. (2022) proposed a content-aware privacy protection method (CPP) that considered the content attribute. Specifically, the CPP method was based on using k-anonymity to generate dummy content attributes to protect the private content. But it was not enough to resist privacy intrusion. Wu et al. (2020) proposed a location privacy-preserving system for RLBS by constructing “cover-up ranges” to protect the query ranges associated with a location query sequence. However, it had always been inefficient. Khan et al. (2021) and Li et al. (2022) adopted suppression method to cut off the correlation between user location and time to protect the trajectory privacy of users. In this method, k tasks were selected from n tasks completed by the user and uploaded to the server in an out-of-order combination (Nie et al., 2021; Khan et al., 2022). Although the trajectory privacy of users was protected, the sensing data lost relevance with time, and the data were meaningless. In addition, this method needed to suppress n-k pieces of sensing data in any case, which would also cause the decline of service quality (Shafiq et al., 2020b; Xu et al., 2021).

Although the above strategies protect users' location privacy in the crowdsensing environment, they failed to take into account the availability of crowdsensing task data and the operation of incentive mechanism. To solve the above problems, this paper proposes a privacy protection strategy based on Q-learning particle swarm optimization algorithm. By generalizing tasks, this new algorithm makes the attacker unable to distinguish the specific tasks completed by users, cuts off the association between users and tasks, and protects users' location privacy.

## Task characteristics and system model

This section detailed introduces the characteristics of crowdsensing task and the proposed privacy protection system model for these characteristics.

### Task characteristics

Crowdsensing task has four characteristics: authenticity, position sensitivity, time sensitivity, and delay tolerance.

1) Authenticity

The data collected by crowdsensing task must be true and valid.

2) Location sensitivity

The location where data are generated is an important attribute. Users can only perceive data near the location required by crowdsensing tasks. The task with stronger location sensitivity has a smaller collection range.

3) Time sensitivity

Data are closely related to its generation time, and different tasks have different time sensitivities to data. Crowdsensing tasks require users to complete within a certain perception period, and the granularity of the perception period is set according to the time sensitivity.

4) Delay tolerance

Most crowdsensing tasks are only sensitive to the time of data generation, but not to the time of data use, that is, crowdsensing tasks are allowed to delay submission. Different tasks have different delay tolerance, and the allowable delay submission duration is different.

### System model

**Figure 1:**
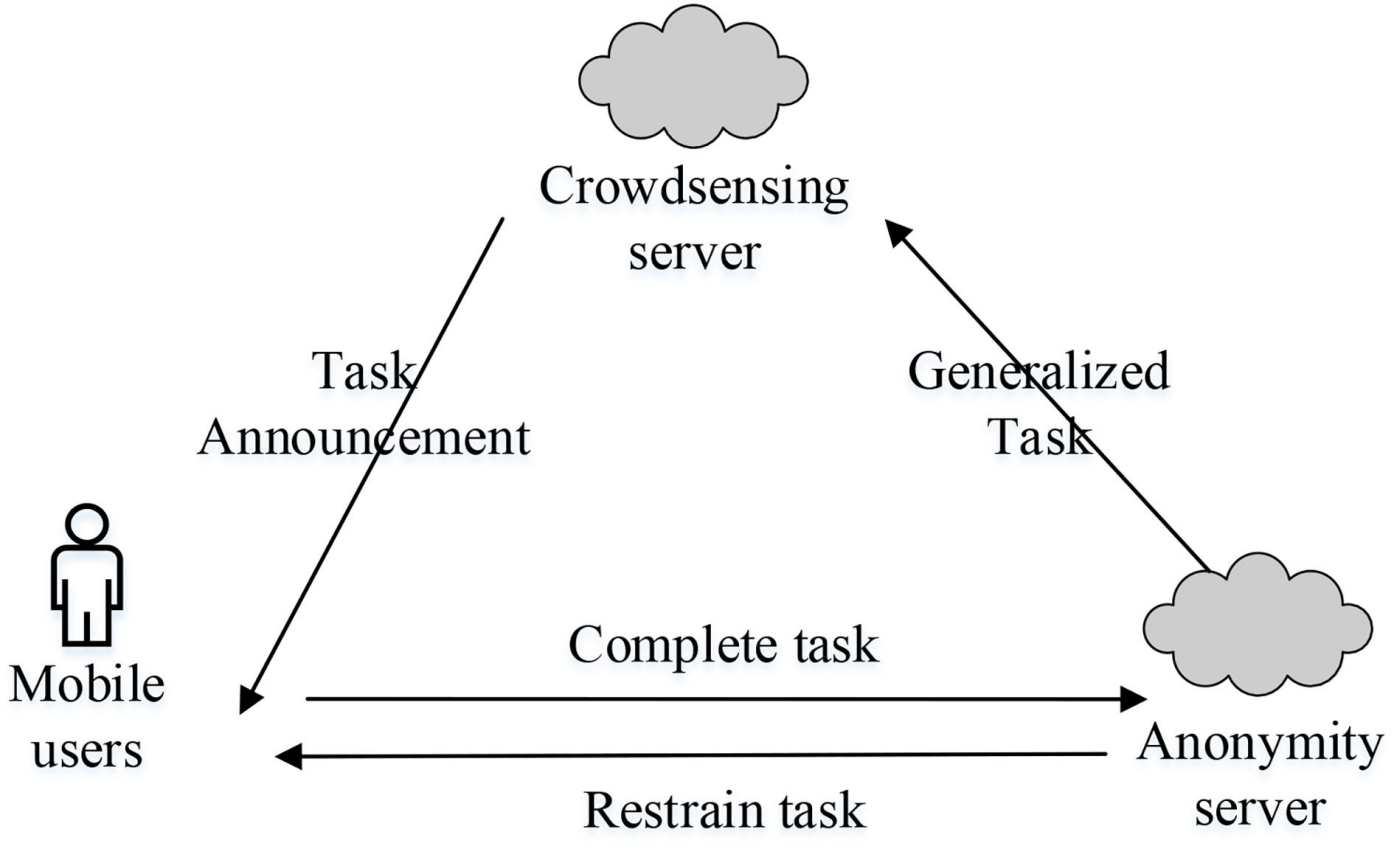
System framework.

The overall proposed system model mainly includes three interaction topics: mobile users, anonymous server, and crowdsensing server as shown in Figure 1.

Mobile user: mobile user *u* is the finisher of the crowdsensing task. They accept the task released by the crowdsensing server and go to task location *l* within the specified sensing period *t*_1_. After the sensing task data *d*, they send the task *Task* = {*u, d, l, t*_1_} to the anonymous server.

Task announcement: These tasks are published by the crowdsensing server. The mobile user then completes these tasks.

Anonymous server: anonymous server is the protector of user privacy. After receiving the task completed by mobile users, the anonymous server will store the task data to the last sensing period *t*_*n*_ before the task submission time limit (i.e., after the end of *t*_*n*_ period, the data collected in *t*_1_ period may become invalid). It selects tasks completed by other *k*−1 users in *t*_1_ period to form anonymous dataset *S* = {< *d*_1_, *l*_1_>,< *d*_2_, *l*_2_ >, ⋯,< *d*_*n*_, *l*_*n*_ >}. The task data $Task^{\prime }=\left\{u,d,l,t_{1}\right\}$ completed by the user u are generalized to $Task^{\prime }=\left\{u,S,t_{1}\right\}$ and forwarded to the crowdsensing server. If there are not enough candidate confounding tasks for the task's sensing period *t*_1_, the task is suppressed and the user is notified. Through the generalization of tasks, the anonymous server makes the crowdsensing server unable to distinguish which task in k tasks that user u has completed, and thus, it realizes the k-anonymous privacy protection requirement.

Crowdsensing server: crowdsensing server is the consumer of sensing data, and it is responsible for releasing tasks to mobile users. Each published task includes the following data: data type (such as temperature, air pressure, speed, etc.), data sensing range *l* (the size is defined according to position sensitivity), data sensing period *t*_1_ (the length is defined according to time sensitivity), and submission time period *t*_*n*_ (it is defined according to delay tolerance). The crowdsensing server only needs to extract {*d, l, t*_1_} from the generalized task dataset S before time period *t*_*n*+1_, and reward user *u* according to the incentive mechanism. It does not need to pay attention to which specific task data are collected by which user, nor does it need to submit data immediately after completing the task.

## Task decision problem for minimizing inhibition rate

In this paper, if there are not enough candidate confounding tasks in the privacy protection mechanism, the target task will be suppressed. Too high inhibition rate will reduce the enthusiasm of users to participate in the application of crowdsensing and also affect the efficiency of the crowdsensing server to collect data. How to minimize the inhibition rate under the privacy protection requirements of k-anonymity is a core problem.

### Task suppression analysis

To reduce the suppression rate, it is necessary to first identify the circumstances under which task submission should be suppressed. This section summarizes three situations in which suppression tasks are required.

1) The sensing period of the task is less than other *k*−1 users' sensing data. At this time, there are too few users who complete the task, and there are not enough users to cooperate to generalize the task. Therefore, the crowdsensing server needs to make use of appropriate incentive mechanism to improve the user participation rate (Yang et al., 2016; Wang et al., 2020).
2) When a user continuously completes a task, an attacker with background knowledge can combine the user's last and next completed tasks for analysis and utilize the maximum movement speed attack model (Wang et al., 2017) to exclude a large number of confused tasks, thus destroying k-anonymity. Therefore, not all other tasks in the same sensing period are effective confounding tasks. When the number of effective confounding tasks is less than *k*−1, the tasks should be suppressed.
3) There are no less than *k*−1 effective confusion tasks. According to the analysis of the user's generalized task set based on the maximum movement speed attack, it can be known that the submission of each task and the selection of confusion task will affect the number of effective confusion tasks of the next task. Therefore, if a certain task is submitted, it may lead to fewer confused tasks for subsequent multiple tasks, thus greatly improving the overall rejection rate, and this task should also be suppressed.

### Anonymous sets and candidate sets

It can be seen from the previous section that whether a task needs to be suppressed is related to the effective confusion task and the generalized task set information. We define the set of effective confusion tasks as the candidate set and the generalized task set as the candidate set. To facilitate calculation, task location *l* is used to represent the corresponding task, and the specific definitions are as follows.

Definition 1. (Candidate set) For any *l*_*i*_ ∈ *C*_*i*_, which is indistinguishable from the task *l*_*u,i*_ completed by user u at time period *t*_*i*_, *C*_*i*_ is called the candidate set of user u at time period *t*_*i*_.

Definition 2. (Anonymous set) $A_{i}=\underset{m=1}{\overset{k-1}{\cap }}\left\{l_{m}\right\}\cap \left\{l_{u,i}\right\}$, where any *l*_*m*_ ∈ *C*_*i*_ is a confusable task and *l*_*u,i*_ is the real task, then *A*_*i*_ is called an anonymous set of user u in time period *t*_*i*_. When the number of task positions in candidate set *C*_*i*_ is less than *k*−1, anonymous set *A*_*i*_ cannot contain *k*−1 confusion task positions, so the task is suppressed. We express the relationship among inhibition rate, candidate set, and anonymous set as follows:


(1) $$\begin{array}{l} Minimize\;:\;\mu =P\left(|C_{i}|\leq k-1\right) \end{array}$$

(2) $$\begin{array}{l} Subject\;to\;:\;|\;A_{i}|=k \end{array}$$

The selection of anonymous set will affect the candidate set in the next period. The problem of minimizing the inhibition rate is transformed into selecting an anonymous set *A*_1_, so that the possible candidate set sequence (*C*_1_, *C*_2_, ⋯, *C*_*n*_) has the least candidate set less than *k*−1 confusion task. This section introduces the relationship and calculation method of candidate set and anonymous set in detail.

A. Candidate set

Because the crowdsensing task has authenticity, time sensitivity, and location sensitivity, the confusion task in the candidate set cannot use fake data or historical data, but only real data were collected by other users in the same sensing period *t*_*i*_. Considering the maximum speed attack model, it needs to make the task of candidate set and the completed task of goal user *u*_0_ indistinguishable. Not all tasks performed by *t*_*i*_ have a confounding effect (Mutalemwa and Shin, 2020; Jin et al., 2021). Therefore, the calculation of valid candidate set *C*_*i*_ needs to consider the anonymous set *A*_*i*−1_ of the previous period and the task position *l*_*u,i*+1_ of the task completed by user *u*_0_ in the next period *t*_*i*+1_.

*t*_*n*_ is the submission time limit of the task completed by user *u*_0_ in time period *t*_1_. When n = 1, it indicates that the user has completed only one task before the task submission time limit, and the trajectory privacy of continuous task submission does not need to be considered. Therefore, except for the tasks completed by target users themselves, all tasks completed in time period *t*_1_ can be added to the candidate set as confusion tasks. Formula (3) calculates the candidate set in the case of n = 1, where *L*_1_ represents the task location set of all users in time period *t*_1_. The candidate set cannot include tasks of target user *u*_0_.


(3) $$\begin{array}{l} C_{1}=L_{1}/\left\{l_{u_{0},1}\right\} \end{array}$$

When *n* ≥ 2, it needs to protect the privacy of the user's trajectory. Candidate set *C*_*i*_ is calculated according to different formulas of *i*. When *i* = 1, it indicates that the user has just started to submit tasks. It needs to ensure that all tasks in the candidate set can reach the real task location completed by user *u*_0_ in the user period *t*_2_. The candidate set in this case is calculated by formula (4), where function *L*(*l, r, t*) represents the task set of all users in time period t in a circular region with location *l* as the center and *r* as the radius. In formula (4), the center of the circle is the location of the real task completed by the user *u*_0_ in time period *t*_2_, the radius is the maximum distance moved by user from *t*_1_ to *t*_2_, and the time period of confusion task is *t*_1_.


(4) $$\begin{array}{l} C_{1}=L\left(l_{u_{0},2},v_{m}\times \left(t_{2}-t_{1}\right),t_{1}\right)/\left\{I_{u_{0},1}\right\} \end{array}$$

When *i* = 2, 3, ⋯, *n* − 1, a reasonable candidate set *C*_*i*_ must ensure that all tasks in anonymous set *A*_*i*−1_ in time period *t*_*i*−1_ move with the maximum speed *V*_*m*_ starting from time period *t*_*i*−1_ and it can reach any confusion task position in *C*_*i*_ within time period *t*_*i*_. It should also ensure that starting from the confusion task position in *C*_*i*_, it can reach the position *l*_*u,i*+1_ of the real task completed by the user in that period within *t*_*i*+1_. Formula (5) computes the candidate set in this case, where *l*_*u*_*j*_,*i*−1_ ∈ *A*_*i*−1_.


(5) $$\begin{array}{l} C_{i}=\underset{j=0}{\overset{k-1}{\cap }}L(l_{u_{j},i-1},v_{m}\times (t_{i}-t_{i-1}),t_{i})\cap L(l_{u_{0},i+1},v_{m}\\ \;\times (t_{i+1}-t_{i}),t_{i})/\{l_{u_{0},i}\} \end{array}$$

When *i* = *n*, no tasks in time *t*_*i*+1_ need to be reachable. Therefore, we only need to consider the reachability of the confusion task in anonymous set *A*_*i*−1_ and candidate set *C*_*n*_. Formula (6) computes the candidate set for this case, where *l*_*u*_*j*_,*n*−1_ ∈ *A*_*n*−1_.


(6) $$\begin{array}{l} C_{n}=\underset{j=0}{\overset{k-1}{\cap }}L\left(l_{u_{j},n-1},v_{m}\times \left(t_{n}-t_{n-1}\right),t_{n}\right)/\left\{l_{u_{0},n}\right\} \end{array}$$

B. Anonymous set

The anonymous set *A*_*i*_ of user *u*_0_ in time period *t*_*i*_ is composed of *k*−1 confusion task locations selected from *C*_*i*_ and the user's own task locations, as shown in formula (7). where *l*_*m*_ ∈ *C*_*i*_.


(7) $$\begin{array}{l} A_{i}=\underset{m=1}{\overset{k-1}{\cap }}\left\{l_{m}\right\}\cap \left\{l_{u,i}\right\} \end{array}$$

Each candidate set *C*_*i*_ has $C_{\left|C_{i}\right|}^{k-1}$ anonymous set. If a task has less than *k*−1 confusion tasks in candidate set *C*_*i*_, the task needs to be suppressed.

### Complexity analysis

According to the calculation formula of candidate set, we can know that the candidate set of the task submitted in the next period is related to the anonymous set of the current period. The anonymous set is selected from the candidate set, and they influence each other to jointly determine the overall suppression rate of the continuous task submitted by the users. Whether the task is suppressed or how to select the anonymous set should not only consider whether the current task is suppressed, but also the subsequent effects. However, each anonymous set is selected from the candidate set, there are $C_{\left|C_{i}\right|}^{k-1}$ selection methods, and the overall inhibition rate μ of each selection method may be different. Finally, there may be $\overset{n}{\underset{i=1}{\sum }}\left(C_{\left|C_{i}\right|}^{k-1}+1\right)$ selection methods. To find the anonymous set with the lowest overall suppression rate, the time complexity of traversing all possibilities is $O\left(\overset{n}{\underset{i=1}{\sum }}|L_{i}|!\right)$. If *n* is large, or if |*L*_*i*_| is larger, the choice is very large. Therefore, brute force calculation using the exhaustive method is not suitable for solving the problem.

## Task decision-making scheme based on Q-learning particle swarm optimization

In this paper, a Q-learning particle swarm optimization method (QLPSO) is adopted to solve the task decision problem with minimal inhibition rate (Qi et al., 2021). QLPSO is a machine learning method that combines deep learning with particle swarm optimization (PSO) (Shafiq et al., 2020a). The idea is that any state is accessed repeatedly by an agent. It tries different actions to update a neural network. Finally, the neural network can fit an action-return function and output an action that approximates the optimal return according to the agent's current state.

Particle swarm optimization algorithm (PSO) (Yin and Li, 2020) is a heuristic algorithm to simulate the foraging behavior of birds, and it seeks the optimal solution by updating the particle velocity and position. Suppose that the particle position and velocity at time *t* are *x*_*i,t*_ and *v*_*i,t*_, respectively, then the position *x*_*i,t*+1_ and velocity *v*_*i,t*+1_ of particle *i* at time *t*+1 are as follows:


(8) $$\begin{array}{l} v_{i,t+1}=\omega v_{i,t}+r_{1}rand(L_{B,i,t}-x_{i,t})\\ \;+r_{2}rand(G_{B,t}-x_{i,t}) \end{array}$$

(9) $$\begin{array}{l} x_{i,t+1}=x_{i,t}+v_{i,t+1} \end{array}$$

where *rand* is a random number between 0 and 1. *L*_*B,i,t*_ is the historical optimal position of particle *i* at time *t*. *G*_*B,t*_ is the global optimal position of all particles at time *t*. ω, *r*_1_ and *r*_2_ are inertia weight, self-learning factor, and global learning factor, respectively.

ω, *r*_1_, and *r*_2_ are important parameters that affect the optimization performance of the algorithm. In previous studies, Hou et al. (2016) proposed APSO with nonlinear inertia weight reduction. APSO mainly balances the relationship between global optimization and local optimization through adaptive adjustment ω and improves the overall optimization ability of the algorithm. Liu Y. X. et al. (2019) further proposed a Q-learning particle swarm optimization (QLPSO), which realized adaptive control through Q-learning to ω, *r*_1_ and *r*_2_, thus achieving better control effect through adaptive global optimization of the adjustment algorithm. Therefore, QLPSO is chosen to optimize the algorithm in this paper.

**Figure 2:**
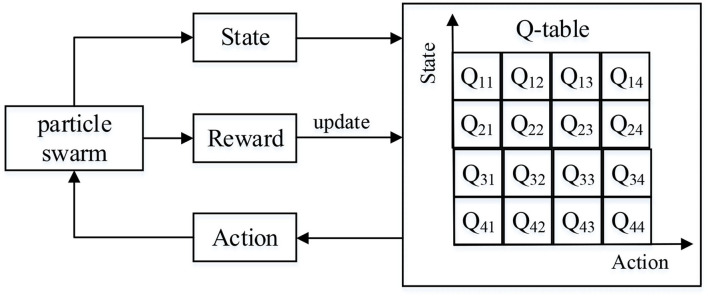
Framework of Q-learning.

Q-learning mainly includes four important parts: state, action, Q table, and reward. Its framework is shown in Figure 2. In here, the Q table is responsible for instructing the agent to select the action with the maximum Q value in a certain state, and it needs to be updated during training.


(10) $$\begin{array}{l} Q_{L}(s_{t+1},a_{t+1})=(1-\alpha )Q_{L}(s_{t},a_{t})+\alpha [R(s_{t},a_{t})\\ \;+\gamma \underset{a}{\max}(Q_{L}(s_{t+1},a))] \end{array}$$

where α is the learning rate. γ is the discount factor. *R*(*s*_*t*_, *a*_*t*_) is the immediate reward of performing action *a*_*t*_ in state *s*_*t*_. *Q*_*L*_(*s*_*t*_, *a*_*t*_) is the cumulative reward at time *t*. *Q*_*L*_(*s*_*t*+1_, *a*_*t*_) is the reward of executing action *a* at time *t* + 1. *Q*_*L*_(*s*_*t*+1_, *a*_*t* + 1_) is the cumulative reward at time *t* + 1. QLPSO designs corresponding strategies from four aspects of state, action, Q table, and reward based on the characteristics of Q-learning and the framework of PSO and finally realizes the adaptive PSO parameter control.

### State and action

According to the characteristics of PSO, the particle state defined in this paper is mainly composed of two parts: decision space state and target space state.

1) Decision space state: according to the distance between the current particle and the global optimal particle position, it can be divided into four states: nearest, near, far, and furthest, marked as *R*_*S,i,t*_ = 1, 2, 3, 4 as shown in Table 1.

**Table 1:** Decision space state.

**Distance**	**Decision space state**	***R*_*S,i,t*_**
0 ≤ *d*_*i*_ ≤ 0.25Δ*D*	Nearest	1
0.25 ≤ *d*_*i*_ ≤ 0.5Δ*D*	Near	2
0.5 ≤ *d*_*i*_ ≤ 0.75Δ*D*	Far	3
0.75 ≤ *d*_*i*_	Furthest	4

In Table 1, *d*_*i*_ is the distance between the position of particle *i* and the globally optimal position of particle. Δ*D* is the maximum distance between all particle positions and the globally optimal particle positions.

2) Target space state: according to the relative performance between the fitness of the current particle and the globally optimal particle and the fitness of the globally worst particle, the four states (minimum, small, large, and maximum) are marked as *R*_*F,i,t*_ = 1, 2, 3, 4 shown in Table 2.

**Table 2:** Target space state.

**Fitness**	**Target space state**	***R*_*F,i,t*_**
0 ≤ *f*_*i*_ ≤ 0.25Δ*F*	Minimum	1
0.25 ≤ *f*_*i*_ ≤ 0.5Δ*F*	Small	2
0.5 ≤ *f*_*i*_ ≤ 0.75Δ*F*	Large	3
0.75 ≤ *f*_*i*_	Maximum	4

In Table 2, *f*_*i*_ is the difference between the fitness of particle *i* and that of the globally optimal particle. Δ*F* is the difference between the fitness of the global worst particle and the fitness of the global best particle.

On the other hand, according to the current particle state and Q-table, the particle will perform two behaviors: global search and local search. Relevant research (Liu Y. X. et al., 2019) shows that when there is a reasonable time allocation between local search and global search, the intelligent algorithm has good robustness. Therefore, the proposed algorithm performs global search behavior in the first 90% of iterations and local search behavior in the last 10% of iterations. Furthermore, the global search behavior is further subdivided into four behaviors: large-range search, small-range search, slow convergence, and fast convergence, marked as *A*_*F,i,t*_ = 1, 2, 3, 4. The above behaviors correspond to a set of ω, *r*_1_, and *r*_2_ parameters (their values are set according to the literature Liu Y. X. et al., 2019), as shown in Table 3.

**Table 3:** Relationship between particle action and parameter value.

**Particle action**		***A*_*F,i,t*_**	**ω**	***r*_1_**	***r*_2_**
Global search	Large-range search	1	1.0	2.5	0.5
	Small-range search	2	0.8	2.0	1.0
	Slow convergence	3	0.5	1.0	2.0
	Fast convergence	4	0.4	0.5	2.5
	Local search		0	0	3.0

### Q table and reward method

The function of Q table is to determine the next action of the particle according to its current state, and the particle state defined in this paper includes decision space state and decision space state. Therefore, the proposed algorithm in this paper designs a 4 × 4 × 4 three-dimensional Q table, and the elements in the table are labeled as *Q*_*T*_(*R*_*S,i,t*_, *R*_*F,i,t*_, *A*_*S,i,t*_). In this paper, the behavior of particles is selected according to the state of particles, and the process of obtaining new particles is as follows. First, the values of *R*_*S,i,t*_ and *R*_*F,i,t*_ are obtained according to the state of the current moment t of particle *i*. Second, it compares *Q*_*T*_(*R*_*S,i,t*_, *R*_*F,i,t*_, 1), *Q*_*T*_(*R*_*S,i,t*_, *R*_*F,i,t*_, 2), *Q*_*T*_(*R*_*S,i,t*_, *R*_*F,i,t*_, 3), and *Q*_*T*_(*R*_*S,i,t*_, *R*_*F,i,t*_, 4). Assume that the maximum value of the four is *Q*_*T*_(*R*_*S,i,t*_, *R*_*F,i,t*_, ε)(ε = 1, 2, 3, 4), then *A*_*F,i,t*_ = ε. It selects the corresponding particle behavior according to Table 3, that is, the parameter values required by the particle update formula [Formulas (8) and (9)]. Finally, the particle *i* at time t of the next iteration is obtained according to the current particle updating formula.

Q table, on the other hand, needs to reward and punish based on the behavior of particles. Therefore, this paper defines the reward method as follows: when the particle performs a certain behavior, if the particle's performance becomes better, the corresponding Q value should be rewarded; otherwise, it should be punished. The corresponding operation is as follows: if according to *A*_*F,i,t*_ = ε, the fitness of the new particle obtained by the corresponding particle updating formula becomes better, then:


(11) $$\begin{array}{l} Q_{T}\left(R_{S,i,t},R_{F,i,t},A_{S,i,t}\right)=Q_{T}\left(R_{S,i,t},R_{F,i,t},A_{S,i,t}\right)+5 \end{array}$$

Otherwise,


(12) $$\begin{array}{l} Q_{T}\left(R_{S,i,t},R_{F,i,t},A_{S,i,t}\right)=Q_{T}\left(R_{S,i,t},R_{F,i,t},A_{S,i,t}\right)-5 \end{array}$$

Since (*s*_*t*_, *a*_*t*_, *r*_*t*_, *s*_*t*+1_) in this paper is a time-dependent sequence, the sample has continuity. If the Q value is updated after obtaining the sample, it will be affected by the sample distribution and the effect will not be good. Therefore, experience replay technology is adopted in QLPSO training to store the data obtained by agents. Then, random sampling is used to break the association between datasets. The QLPSO-based inhibition rate optimization algorithm using experience replay is described as Algorithm 1.

**Algorithm 1:** QLPSO-based inhibition rate optimization with experience replay.

Input: *L*_1_, *L*_2_, ⋯, *L*_*n*_
Output: *A*_1_
Begin
Step 1. Set the parameters of the QLPSO
Step 2. $Q=\overset{n}{\underset{i=0}{\sum }}\theta_{i}\left(s_{i},a_{i}\right)$
Step 3. Initialize the candidate set *C*_1_ of user *u* in time period *t*_1_ as state *s*_1_
Step 4. for episode ← 1 to M
Step 5. Select action *a*_*i*_ according to the policy
Step 6. Perform the action *a*_*i*_ and get a reward *r*
Step 7. Save (*s*_*t*_, *a*_*t*_, *r*_*t*_, *s*_*t*+1_) to experience playback pool D
Step 8. if *len*(*D*) > *OBSERVER* then
Step 9. Extract part of the data from D as the training sample
Step 10. Use training samples to obtain the target Q value
Step 11. Update the Q network with the target and current Q value
end if
end for
Step 12. return $\underset{a}{\max}Q^{*}\left(s,a;\theta \right)$
End

## Experiments and analysis

The experiments are conducted on the public GeoLife GPS Trajectories. The GPS trajectory dataset is collected by the GeoLife project of Microsoft Research Asia, which records the trajectories of 182 users for 3 years (Hu et al., 2019). The following will introduce the experimental environment and parameter settings and then analyze the experimental results.

Table 4 shows the experimental environment. We are using an Intel Core i7 4790 processor, 4-core 8-thread, 3.6GHz, 1060Ti video card, 32GB memory, 16GB RAM, and Ubuntu 6.04 with a 128GB solid-state drive (SSD). It is programmed in Python with TensorFlow and Numpy libraries. First, the datasets are processed, 100 users' data in a specific range are selected, and some crowdsensing tasks are randomly assigned. Crowdsensing tasks are evenly distributed in the square area. It is assumed that the user moves to the vicinity of the task during the task period and completes the task with a certain probability. To improve the reference value of experimental data, we repeat each experiment for five times and take the average value to ensure the generality of experimental results. To reflect the superiority of the scheme in this paper, when the control experiment is needed, we adopt the most common method of generalized user privacy protection scheme as the baseline and compare it with the QLPSO algorithm in this paper.

**Table 4:** Experimental environment.

**Parameter**	**Values**
Processor	Intel Core i7 4790 @3.6 GHz
Memory	32 GB
Video card	1060 Ti
RAM	16 GB
Solid-state drive (SSD)	128 GB
System	Ubuntu 6.04
Programming language	Python

Basic parameter settings are shown in Table 5. The total number of training fragments M and learning parameter α is selected according to the experimental results in Subsection Parameter verification experiment in QLPSO. As long as the value of inhibition rate is given priority, the reward coefficient is set as 10^−3^ in this paper. Parameters such as the number of users in the anonymous area, the number of tasks in the area, and the user completion rate in each period are set according to the common scenarios of crowdsensing application. The size of anonymous set is an average value set according to the user's sensitivity to privacy. The action selection probability ε is set as $1/\sqrt{t}$ according to the research in Bloembergen et al. (2015), which can ensure that the proportion of utilization will be higher and higher with the increase of training times.

**Table 5:** Experimental parameters.

**Parameter**	**Value**
Total number of training fragments M	3,000
Number of tasks submitted by users before the submission deadline n	10
Action selection probability ε	$1/\sqrt{t}$
Learning parameter α	0.02
Reward coefficient δ	10^−3^
Discount factor γ	0.8
Number of users in an anonymous zone	130
User task completion rate per period of time	0.9
Number of anonymous area tasks	255
Anonymous set size *k*	6
Task area size	4,000 m × 4,000 m

### Parameter verification experiment in QLPSO

The QLPSO algorithm needs to create a neural network to reduce the inhibition rate and use this network to replace the Q value table in traditional Q-leaning. We use the TensorFlow library to create a three-layer neural network (Shafiq et al., 2020b; Laghari et al., 2021; Sp et al., 2021). The parameter debugging experiment of QLPSO network is introduced below.

Table 6 shows the results of task inhibition rates under different learning rates. It can be seen that when the learning rate is >0.04, it cannot stably converge to a lower inhibition rate, and there are many outliers. The reason is that when the learning rate is too high, the algorithm relies too much on past experience. The probability of exploring unselected anonymous sets is greatly reduced and local convergence is easy to enter. Therefore, according to the experimental results, to reduce the training times and improve the calculation speed without falling into local convergence of the inhibition rate, 0.04 is selected as the learning rate in this paper.

**Table 6:** The effect of learning rate on average inhibition rate (AIR) (%).

Learning rate	0.02	0.03	0.04	0.05	0.06
AIR	10.2	10.2	10.2	15.3	15.6

Table 7 shows the influence of training times on the average reward value when the learning rate is 0.04, where OPT values are the inhibition rate under the optimal decision obtained through brute force exhaustive exertion. In practice, the time complexity of exhaustive method is too high and the calculation speed is too slow. According to the formula of reward value, the higher average return value denotes the lower final inhibition rate and the better service quality. The experimental results show that the reward value of sample data tends to converge after 3,000 training times and is very close to the optimal solution. Therefore, when the learning rate is 0.04, 3,000 training times can calculate the anonymous set close to the optimal inhibition rate.

**Table 7:** The effect of training number on average reward value (%).

Episode M	1,000	2,000	3,000	4,000
OPT	92.5	92.5	92.5	92.5
QLPSO	70.2	83.6	86.7	91.6

### The effect of user number on inhibition rate

**Figure 3:**
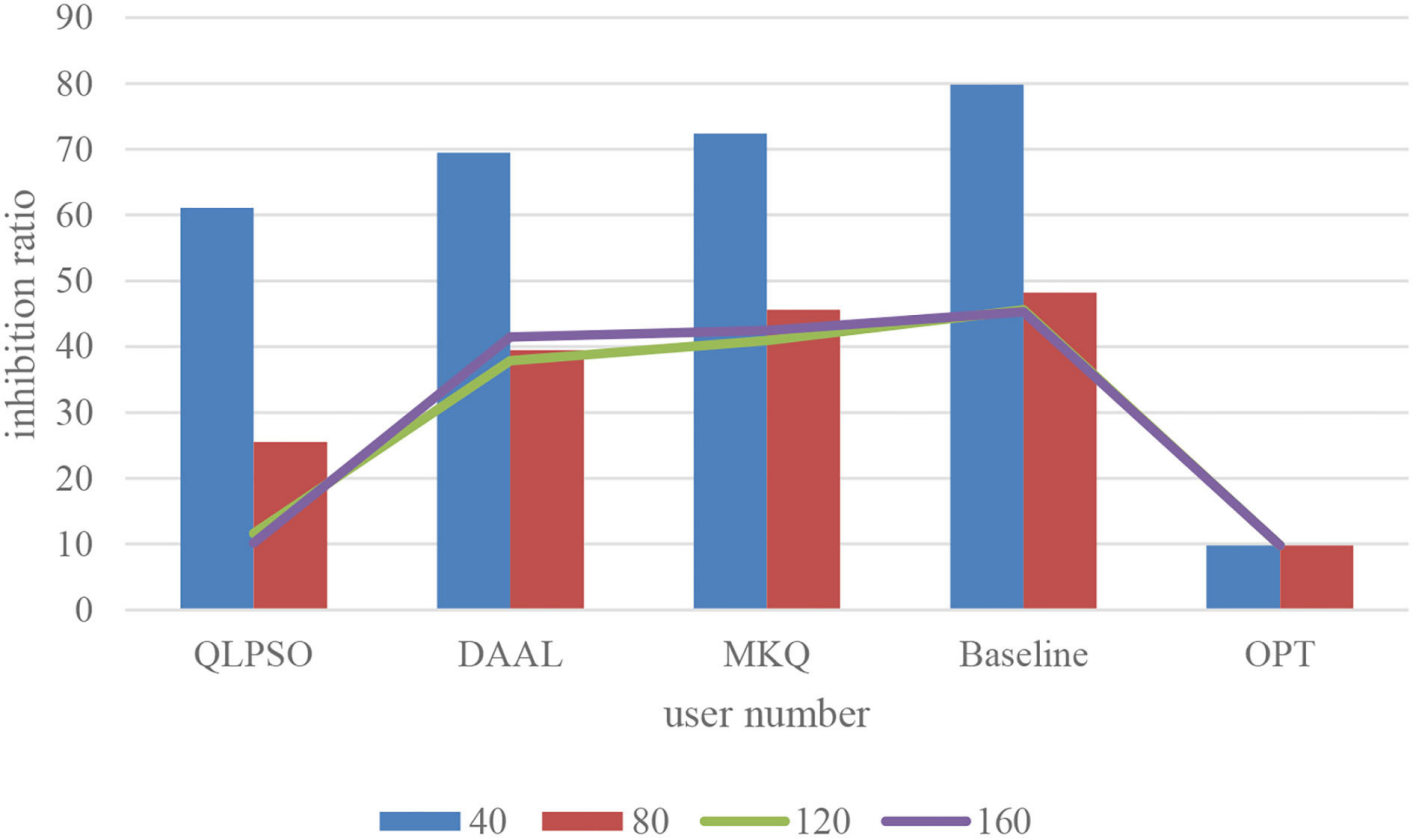
Effect of user number on inhibition rate.

The number of users is the basis for the operation of crowdsensing applications and also an important factor for the success of the privacy protection mechanism of crowdsensing collaboration. In this section, the relationship between the number of users and the inhibition rate is tested under the privacy protection requirement of *k* = 6. The experiment compares the proposed algorithm (QLPSO), the traditional generalized user selection algorithm (baseline), and the optimal inhibition rate (OPT). We also select two methods to make a comparison containing MKQ (Zhang et al., 2020) and DAAL (Zeng et al., 2021). Figure 3 describes the influence of the user number on the inhibition rate. It can be seen that QLPSO's inhibition rate is lowest, and they are very high when the number of users is very small, because when the number of users is small, it is difficult for any scheme to find enough users to participate in the generalization, and the task will be greatly suppressed. So it needs incentive mechanism coordination. Both schemes reduce the inhibition rate when the number of users increases, while the QLPSO algorithm reduces the inhibition rate significantly faster than the baseline and finally approaches the optimal value.

### Effect of delay tolerance on inhibition rate of task

**Figure 4:**
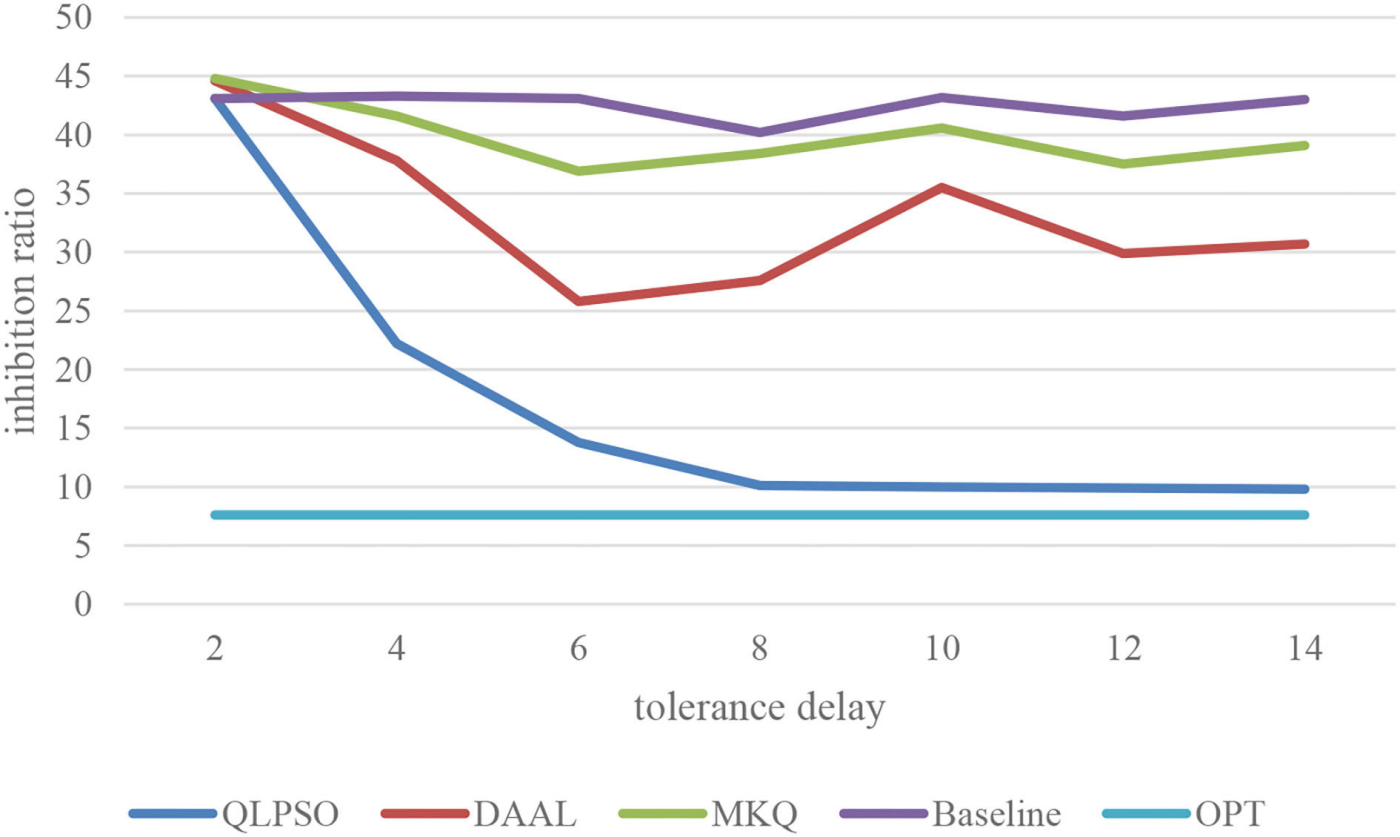
Effect of tolerance delay on inhibition rate.

The tolerance delay affects the optimization space of the QLPSO algorithm for the inhibition rate before the task submission. We test the relationship between tolerance delay and inhibition rate by selecting 100 users to complete the tasks in a specific range. The experimental results are shown in Figure 4, where the *x*-coordinate tolerance delay n represents the number of tasks completed by the users before task failure. It can be seen that when the tolerance delay is 0 or 1, the real-time requirements of tasks are very high. Baseline and QLPSO are both around 42. MKQ and DAAL are both around 45.The reason is that there is no delay tolerance. QLPSO algorithm has no room to optimize the inhibition rate. The inhibition rate of baseline fluctuates around 40–45 when delay tolerance is increased. The inhibition rate of both MKQ and DAAL is declined a lot. Because the inhibition rate of traditional selection method has nothing to do with the tolerance delay of task, the characteristics of the crowdsensing task are not properly utilized. However, the inhibition rate of QLPSO algorithm decreases rapidly with the increase of delay tolerance. When the delay tolerance reaches more than 7, the inhibition rate tends to be stable and is very close to the optimal value. Therefore, the QLPSO algorithm can take advantage of the characteristics of crowdsensing environment and perform under the condition of certain delay tolerance of sensing task.

## Conclusions

This paper proposes a robot privacy protection strategy based on crowdsensing environment. In this paper, we propose a novel location privacy protection based on Q-learning particle swarm optimization algorithm in mobile crowdsensing. Its core ideas are as follows: (1) to ensure the availability of crowdsensing data, (2) to suppress tasks that will destroy user privacy, and (3) to reduce the inhibition rate of tasks. Experimental results show that the proposed strategy performs well in the crowdsensing applications with sufficient delay tolerance and more users' participation. In the future, we will study on the caching mechanism, to reduce the number of interactions between users and the server, to ensure the privacy security of the robot location, and to improve the communication efficiency and apply them in the practical engineering applications.

## Data availability statement

The datasets [GPS trajectory] for this study can be found in the [GeoLife project of Microsoft Research Asia] [https://www.microsoft.com/en-us/download/details.aspx?id=52367].

## Author contributions

DK contributed to the conception of the study and the overall idea of this manuscript. DM performed the robot location privacy protection method design and the data analyses and wrote the manuscript. XC helped to perform the analysis with constructive discussions and collected experimental data. LZ contributed to collecting background information on RBLS. MY contributed significantly to analysis and manuscript preparation. All authors read and approved the final manuscript.

## Funding

This research was funded by the Heilongjiang Provincial Department of Education Science and Technology Innovation Team Construction Project, Grant Number 2019-kyywf-1335, Key R&D Technology Projects in Heilongjiang Province, Grant Number GA21A302, and Basic Scientific Research Project of Heilongjiang Province, Grant Number 2021-KYYWF-0575.

## Conflict of interest

The authors declare that the research was conducted in the absence of any commercial or financial relationships that could be construed as a potential conflict of interest.

## Publisher's note

All claims expressed in this article are solely those of the authors and do not necessarily represent those of their affiliated organizations, or those of the publisher, the editors and the reviewers. Any product that may be evaluated in this article, or claim that may be made by its manufacturer, is not guaranteed or endorsed by the publisher.
